# Supplementary material for: Integration of Proteomic and Metabolomic Data Reveals the Lipid Metabolism Disorder in the Liver of Rats Exposed to Simulated Microgravity
Source: Biomolecules. 2024 Jun 12;14(6):682. doi: 10.3390/biom14060682 (PMC11201887; doi:10.3390/biom14060682)
Supplement: Supplementary file 1 [file biomolecules-14-00682-s001.zip › Supporting Figures.pdf]

Integration of Proteomic and Metabolomic Data Reveals the Lipid Metabolism Disorder in the Liver of Rats Exposed to Simulated Microgravity

Mengyao Ru [1,2], Jun He [3], Yungang Bai [4], Kun Zhang [1,2], Qianqian Shi [2,5], Fang Gao [6], Yunying Wang [4], Baoli Li [7,*] and Lan Shen [2,*]

[1] School of Basic Medicine, Yan’an University, Yan’an 716000, China; rumengyao2022@163.com (M.R.); 18966597676@163.com (K.Z.)
[2] The State Key Laboratory of Cancer Biology, Department of Biochemistry and Molecular Biology, The Fourth Military Medical University, Xi’an 710032, China; sqq15004194219@126.com
[3] Department of Anesthesiology, Xi’an No.3 Hospital, The Affiliated Hospital of Northwest University, Xi’an 710018, China; doctorhejun@163.com
[4] Department of Aerospace Medicine, The Fourth Military Medical University, Xi’an, 710032, China; baiyun_1123@163.com (Y.B.); yunyingwang@fmmu.edu.cn (Y.W.)
[5] School of Life Sciences, Yan’an University, Yan’an 716000, China;
[6] Department of Neurobiology, The Fourth Military Medical University, Xi’an 710032, China; fanggao@fmmu.edu.cn
[7] Yan’an Key Laboratory of Microbial Drug Innovation and Transformation, Yan’an University, Yan’an 716000, China.
[*] Correspondence: lbl_0812@163.com (B.L.); lanshen@fmmu.edu.cn (L.S.), Tel.: +86-29-84774513 (L.S.)

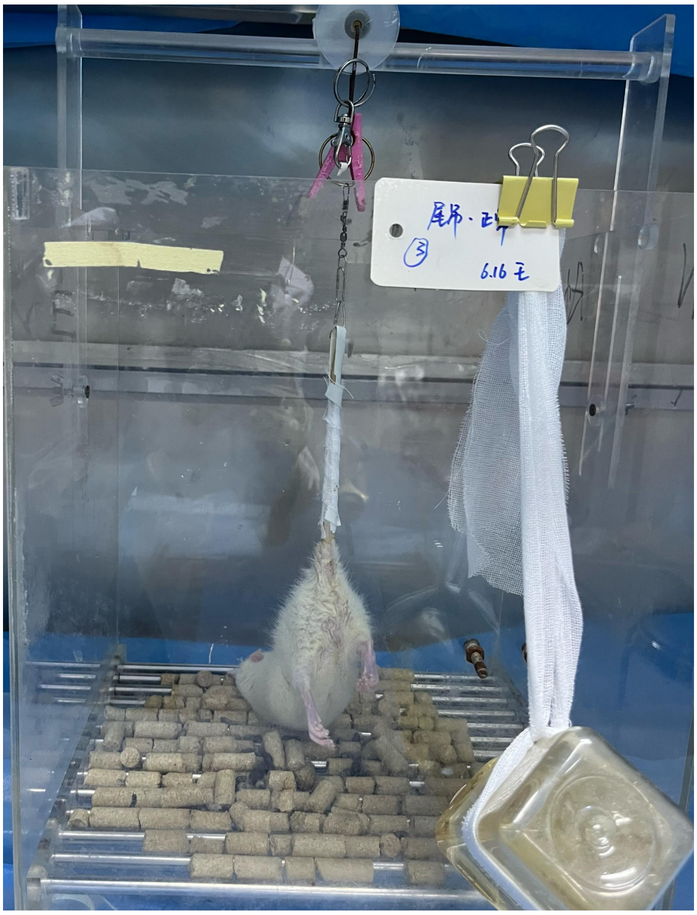

Figure S1: Diagram of rat tail suspension.

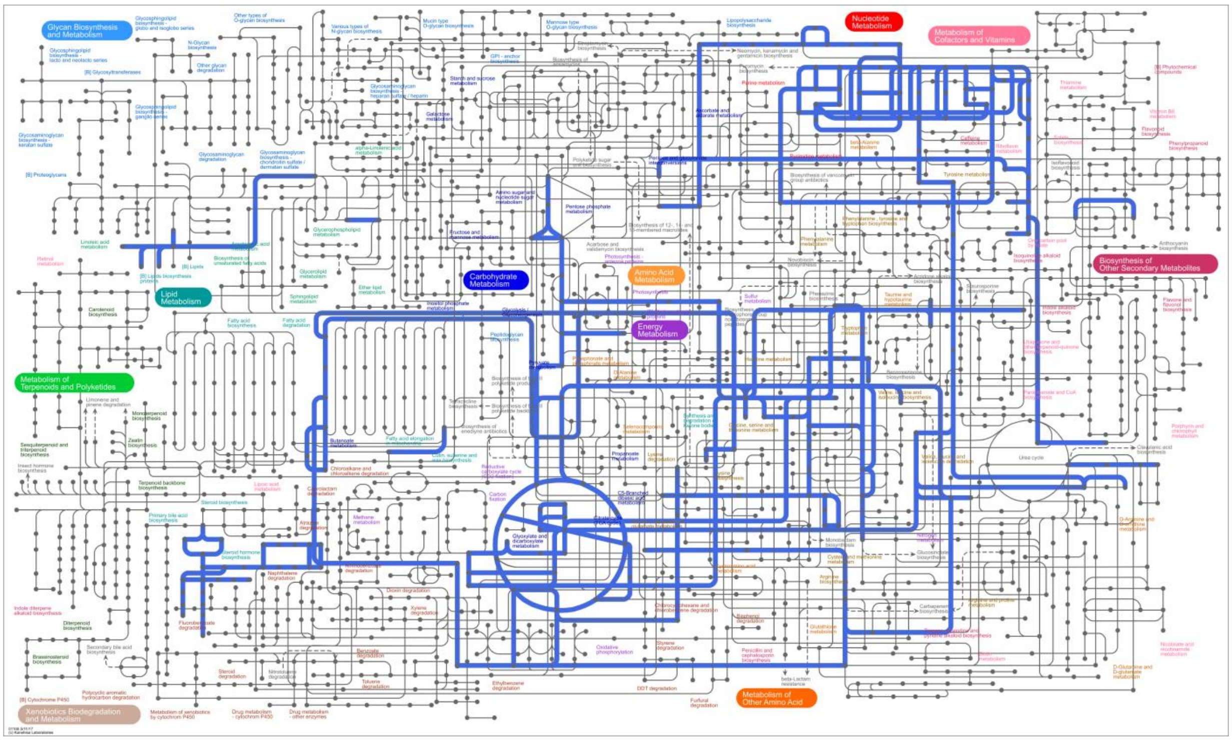

Figure S2: iPath pathway map of co-enrichment pathways.

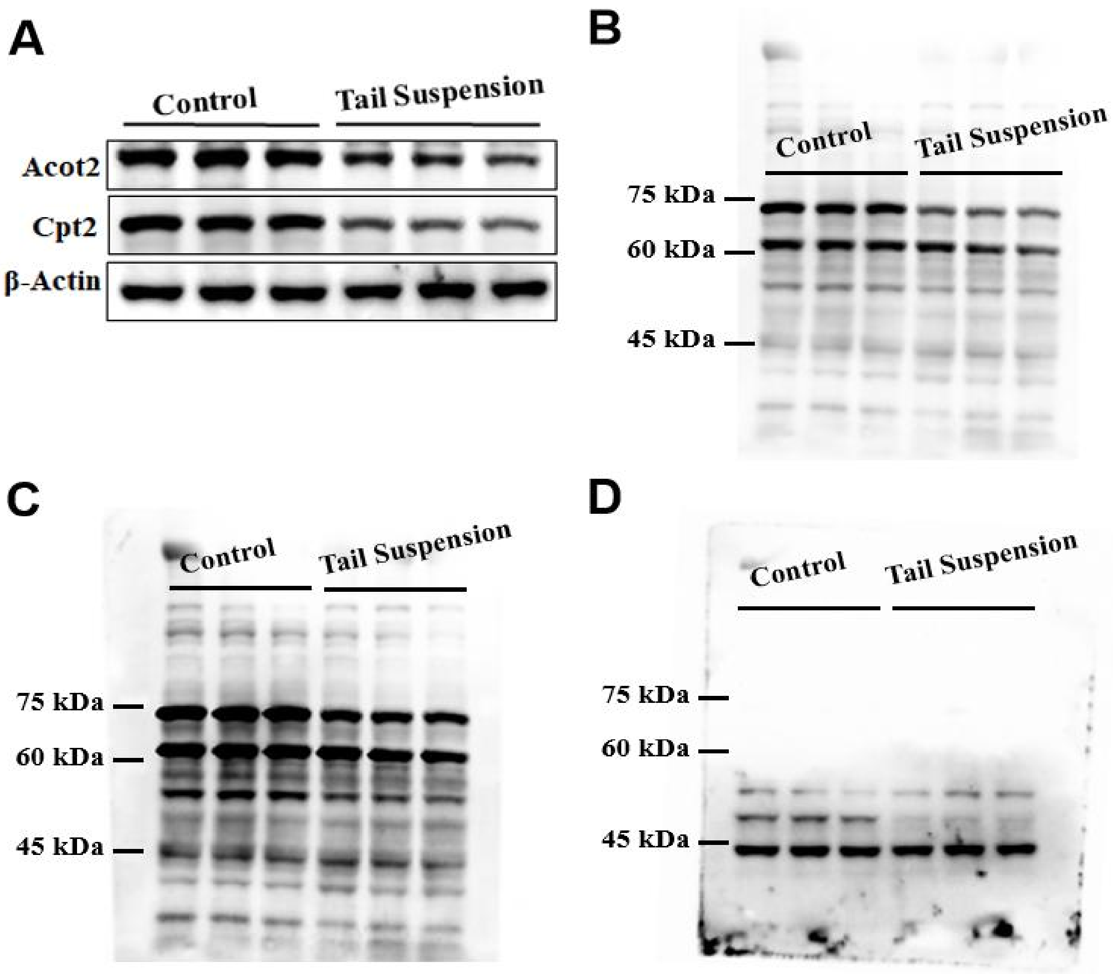

Figure S3: Original Images for Blots. Western blot analysis of molecules whose expression changed significantly in the livers of rats in the control and tail-suspended groups (A). Original image of carnitine palmitoyl transferase 2 (Cpt2) (B), acyl-CoA thioesterase 2 (Acot2) (C), and β-actin (D) in western blot assay.
